# Stem Cell Tracking Technologies for Neurological Regenerative Medicine Purposes

**DOI:** 10.1155/2017/2934149

**Published:** 2017-09-12

**Authors:** Yongtao Zheng, Jiongwei Huang, Tongming Zhu, Ronggang Li, Zhifu Wang, Fukai Ma, Jianhong Zhu

**Affiliations:** ^1^Department of Neurosurgery, Fudan University Huashan Hospital, National Key Laboratory of Medical Neurobiology, The Institutes of Brain Science and the Collaborative Innovation Center for Brain Science, Shanghai Medical College, Fudan University, Shanghai 200040, China; ^2^Department of Urinary Surgery, Zhongshan Hospital of Fudan University, Shanghai, China

## Abstract

The growing field of stem cell therapy is moving toward clinical trials in a variety of applications, particularly for neurological diseases. However, this translation of cell therapies into humans has prompted a need to create innovative and breakthrough methods for stem cell tracing, to explore the migration routes and its reciprocity with microenvironment targets in the body, to monitor and track the outcome after stem cell transplantation therapy, and to track the distribution and cell viability of transplanted cells noninvasively and longitudinally. Recently, a larger number of cell tracking methods in vivo were developed and applied in animals and humans, including magnetic resonance imaging, nuclear medicine imaging, and optical imaging. This review has been intended to summarize the current use of those imaging tools in tracking stem cells, detailing their main features and drawbacks, including image resolution, tissue penetrating depth, and biosafety aspects. Finally, we address that multimodality imaging method will be a more potential tracking tool in the future clinical application.

## 1. Introduction

Neural stem cells (NSCs) are capable of self-renewing, proliferating, and differentiating into cells of the neural lineage, including neurons, astroglia, and oligodendroglia. NSCs had been used as a novel treatment strategy of brain trauma, stroke, and some neurological disorders, such as Parkinson's disease, both in the preclinical experimental and clinical settings [1–3]. As limited control and tracking of endogenous NSCs, exogenous NSCs or neural progenitor cells (NPCs) were used in cell therapy widely. After implanted in damaged brain regions, PSC-derived neurons could reestablish the damaged long range axonal projections and synaptic connections in the host brain [1]. Specifically, fetal brain-derived human neural progenitor cells (hNPCs), which transplanted in the induced injury striatum of an animal model, demonstrate their ability to protect the striatum and improve functional recovery [4]. Overall, neural progenitor/stem cells present a promising therapy strategy in the treatment of various neuronal diseases.

To ensure cell treatments are effective and successful, it is crucial to track the survival, migration, and differentiation of transplanted cells and to track their capabilities of reconstructing brain function and their biological role. Traditional methods to track implanted NSCs such as fluorescence imaging need to kill animals to test whether the transplanted stem cells survive or differentiate into tissue cells [5]. This translation of cell therapies into clinical settings has prompted a need to track the spatial destination, migration pathway, and final distribution of transplanted cells in vivo longitudinally, noninvasively, and repeatedly. Additionally, the advantages of an ideal imaging modality were as follows: high sensitivity of imaging agent, able to image deep tissues, high resolution, tracking transplanted cells for a long time, and very fast image acquisition [6, 7]. Among the various cell imaging modalities, MRI plays an important role in the procedure of transferring cell therapies from the animal experiments to the clinical settings, because of its characteristic of noninvasive and good tissue contrast. These methods have had varying success, and they each have their own strengths and weaknesses of applicability in the central nervous system. For example, PET is a high-sensitive tracking method; however, it also has some limitations: low spatial resolution, radiation exposure, and short-term signal production. Optical imaging, which can track stem cells for a long time without radiation, is not feasible for clinic application as the limited penetration depth and low spatial resolution (Table 1). Therefore, a noninvasive method of tracking stem cells for a long time in the human body is a crucial step before translating stem cell research into clinical application.

In this review, we describe recent advances in the development of novel imaging sensors and tools in the field of tracking stem cells, as well as the benefits and drawbacks of each approach. We will address image spatial/temporal resolution, signal sensitivity, and tracking stem cells for a long time, as well as tissue penetrating depth associated with those imaging technique. Finally, we also describe multimodality molecular imaging of NSC transplantation in consideration that each technique has advantages and disadvantages.

### 1.1. Magnetic Resonance Imaging (MRI)

**Figure 1:**
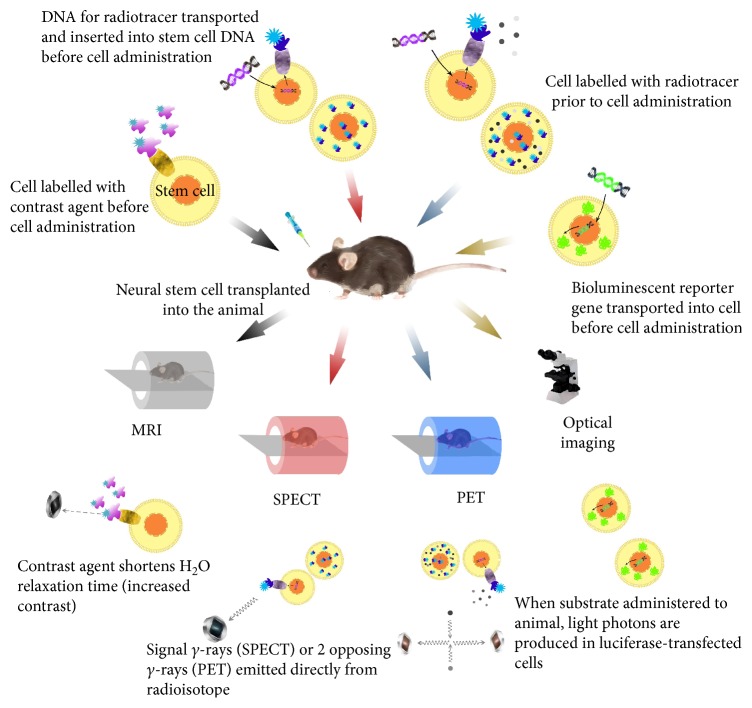
Principles of stem cell labelling for different imaging modalities.

Recently, MRI has become a very important method for real-time, noninvasive tracking stem cell fasting in clinical cell therapy trials, providing high resolution in the field of neurology [8]. The first study of MR tracking of transplanted progenitor cells in the CNS was reported in 1992, in which superparamagnetic contrast agents were used for cell imaging in rat brain [9]. MRI is a well-defined noninvasive cell imaging technique, which has many valuable advantages, for example, it is able to provide an excellent image quality and high sensitivity and spatial 3D resolution, identify labeled cells in their anatomical context, get additional information about the surrounding milieu, and promise clinical applicability with nontoxicity and noninversion (Figure 1).

Gadolinium (III) (Gd^3+^) is a heavy metal contrast agent widely used in clinical and animal experimental MRI. The contrast-enhanced lesions or labeled transplanted stem cells will appear as hyper intense on T1-weighted and hypointense on T2-weighted images, as Gd^3+^-based contrast could shorten T1 and T2 relaxation times. Therefore, those Gd^3+^-based agents were called T1 agents. However, because of their low uptake by cells, many tracking methods, such as coupling of the contrast agent to a membrane translocation peptide or using transfection agents during the process of transfection, are available for increasing the uptake rate of the contrast agent [10]. Next to gadolinium, manganese is another potentially useful “positive” T1 contrast agent which was used widely to study the function of the brain. As its similar ionic property to Ca^2+^, Mn^2+^ can be taken up by excitable cells of the brain and spinal cord via voltage-gated Ca^2+^ channels and the sodium (Na^+^)/Ca^2+^ exchanger. Also, Mn^2+^ can enter the stem cells through binding with Ca^2+^- and Mg^2+^-binding sites on specific proteins and nucleic acids [11]. In general, manganese is a particularly attractive contrast agent for MRI of the brain to study neuronal activity, to monitor neuronal tracts, and to detect transplanted cell functions, as its property of entering the cell conveniently.

**Figure 2:**
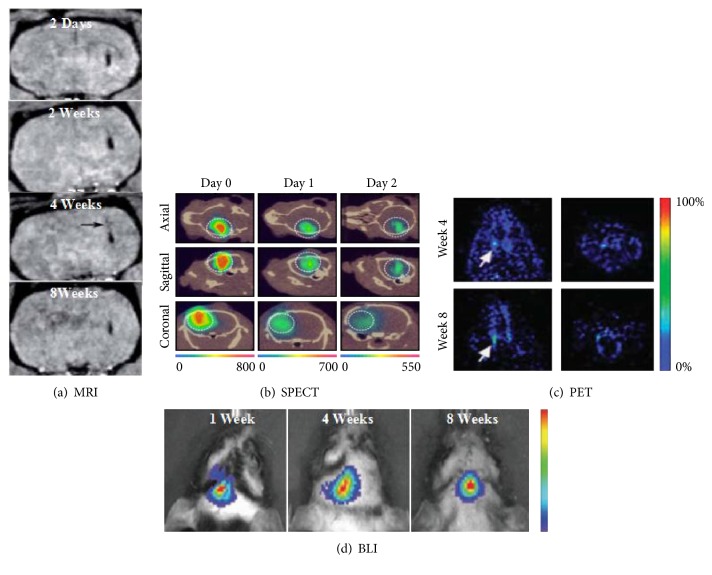
Comparison of imaging techniques for transplanted therapeutic neural stem cells (NSCs). (a) Monitoring of magnetic nanoparticle-labelled NSCs in rat brain using magnetic resonance imaging (MRI). MRI was performed 2 days, 2 weeks, 4 weeks, and 8 weeks after cell transplantation [7]. (b) Single photo emission computed tomography (SPECT) imaging of mouse brain after intracerebral delivery of NSCs loaded with ^111^In. SPECT was performed immediately, 1 day and 2 days after cell transplantation [28]. (c) 9-(4-[^18^F]fluoro-3hydroxymethylbutyl) guanine ([^18^F]FHBG)-labeled embryonic stem cell-derived neural stem cells (NSCs) viewed through positron emission tomography (PET) can be seen localizing in the striatal region of the forebrain [6]. (d) Luciferase photon emission detected through bioluminescence imaging (BLI) 1 week to 8 weeks after transplanting of neural progenitor cells (NPCs) [41].

Over the past decades, iron oxide particles have been developed for more efficient intracellular labeling, due to their high sensitivity, biocompatibility, and increased paramagnetic per mole of metal compared to manganese or gadolinium. These iron oxide particles act locally to reduce the T2 relaxation via inducing strong field inhomogeneity. When T2-weighted pulse sequences were released, these particles will produce a hypointense or signals on the MRI, allowing to catch the vision of the labeled, transplanted cells. As for the experimental model, after transplanted into adult murine brains, MRI could visualize the migration routine of SPIO-labeled stem cells. The study found that SPIO nanoparticle labeling has no adverse effect on the cell survival, proliferation, self-renewal, and multipotency [12]. The two formally approved iron-oxide-based agents used for stem cell labelling, SPIO nanoparticles coated with dextran or low molecular weight carboxydextran, were subsequently removed from the market in 2009 because of economic considerations. As the example of their clinical use, Zhu et al. reported a case of labeling NSCs with SPIO and tracking their survival, migration, and distribution in a patient with brain trauma in the left temporal lobe. The patient was then imaged with MRI weekly for 10 weeks after transplantation. Marked MRI image of transplanted stem cells was observed in vitro noninvasive [13]. More importantly, many studies have reported that SPIO labeling does not affect the function of stem cells and that tracking effect keeps as long as several weeks (Figure 2(a)) [7, 14].

However, there are several limitations in labeling stem cells with magnetic contrast agents. The label will be diluted due to stem cells continuing to proliferate with a fast speed after transplantations. Therefore, the MR signal will decrease even lost over time because of cellular proliferation. Additionally, SPIO nanoparticles will deposit in extracellular tissues when the dead transplanted cells were engulfed by immune cells, such as microglia in the central nervous system, which can lead to a false signal on MRI [15–17]. Although MRI has a unique advantage in tracing the location of stem cells, it cannot reflect the survival state of stem cells and the changes of microenvironment.

In 2005, a new medical imaging technology, named magnetic particle imaging (MPI), was introduced to track transplanted cells with the advantage of imaging the SPION distribution directly and producing linearly quantitative images of SPIO-labeled cells [18]. Since biological tissue itself does not produce an MPI signal, MPI images are extremely sensitive with a high signal-to-noise ratio [19, 20]. Theoretically, MPI is sensitive enough to image 1 pg Fe, meaning this tool has potential to detect even a single stem cell. Importantly, the MPI signal was linear with iron concentration and cell number, which allows for proper cell quantification. As the SPIO tracers are detected directly with MPI, their quantification is simple and straightforward [21]. This is somewhat analogous to fluorine-19 (^19^F) MRI, which can overcome the disadvantages of cell quantification and ambiguity of contrast assignment when used to track stem cells [22]. The directly proportional and linear relationship between the signal strength and concentration of the ^19^F allows quantification of ^19^F-labeled stem cells in vivo [23]. Importantly, ^19^F signal can be overlaid on 1H MR image with a very high quantitative tracking of labeled transplanted cells in vivo because the host tissue is absent in the level of background ^19^F signal. Particularly, compared with hydrogen, ^19^F has a nuclear magnetic resonance sensitivity of 83%, which is suitable for labeling cells [24]. Therefore, it is of high sensitive to use ^19^F MRI for tracking stem cells. In contrast with a diluting process of SPIOs as stem cell proliferation, ^19^F MRI could monitor the spatial-temporal migration dynamic routine of NSCs transplanted into the central nervous system, with the ability of detecting as low as several cells with a considerable high spatial resolution; even the interest labeling cells migrate within an even small scale.

Recently, more clinical grade studies are needed to overcome some limitations of existing MR cell imaging methods. For instance, MR reporter genes were introduced for stable, robust, and long-lasting tracking of the migration of implanted (stem) cells which does not diminish or decrease along with cell division that was the major limitation of the present MR imaging techniques by using routine contrast agents [25]. Also, transgenic cell lines with inbuilt contrast agents were proposed for stem cell transplantation. In addition to develop more sensitive novel contrast agents, increased resolution is also achieved through various means. The most common method includes increasing the number of coil receiver channels, the magnetic field strength, and image acquisition times. In general, equipping various (stem) cell therapy modalities with noninvasive MR imaging techniques has a great potential for clinic application.

### 1.2. Nuclear Medicine Imaging

Nuclear medicine imaging techniques, both PET and SPECT, represent another promising imaging modality to track stem cells which have been used in experimental and clinic trials widely (Figure 1). Before the stem cells were transplanted into the host, a radiotracer, such as ^11^C, ^13^N, ^15^O, and ^18^F, is necessary to label the stem cells in order to detect the transplanted cells though PET/SPECT scanner [26]. The emitted positron from radioisotopes will lose its kinetic energy rapidly while traveling through the surrounding tissue and then interact with electrons resulting in the emission of two high-energy photons of 511 keVat (high-frequency photons) travelling at nearly opposite directions. The PET camera can detect and image these photons in the scanner. SPECT is very similar to PET in its use of radioactive tracer and detection of gamma rays. In general, the operational principle of SPECT is similar to PET; however, what the SPECT scanner detected is signal gamma rays emitted by isotopes. Compared with SPECT, the key characteristic of PET imaging is high sensitivity and temporal resolution. However, a marked advantage of imaging two different radioisotopes at the same time made SPECT an important tracking method. In both techniques, because of their intrinsic tomographic nature, they can present the distribution of labeled stem cells by generating three dimensional images. These images can be used to assess biological features of labelled stem cells, such as blood perfusion, metabolism, and enzymatic activity.

^111^In oxyquinoline, an FDA-approved radiotracer, has been used to image the accumulation and biodistribution of stem cells/progenitor cells in animal models successfully in the previous studies [27]. Due to the lipophilic nature of the ^111^In-oxinemolecule, it can “enter” the cell easily by passively diffusing into the cell membrane. It is possible to image the cells as long as 2 weeks after injection because of the long half-life of ^111^In (2.8 days). Cheng et al. reported that ^111^In-mesoporous silica nanoparticle (MSN) complex shows minimal toxicity to stability and biological activity of NSCs both in vitro and in vivo (Figure 2(b)) [28]. In a rat model of middle cerebral artery occlusion and the controls, cell detection was performed at once and 24 hours after the cell transplantation with a SPECT/CT device. The result showed that as low as 1000 ^111^In-oxine-labeled cells can be detected by the SPECT/CT device; more importantly, the cell viability was not affected by the agents [29]. Besides ^111^In-oxine, another radiolabel agent, ^99^mTc-HMPAO (hexamethylpropylene amine oxime) with a half-life of 6 h, which could avoid the issue of radiation damage, has been used mainly for the stem cell tracking showing low toxicity. In contrast to ^111^In-oxine, proliferating and differentiating abilities of both human and rat MSCs were not affected by ^99^mTc-HMPAO labeling. However, in one study of Gleave et al., labeling neural stem and progenitor cells with ^99^mTc decreased the proliferative capacity of those cells. Clinical studies using ^99^mTc-HMPAO tracking stem cells are mainly involve in those with chronic ischemic cardiomyopathy or myocardial infarction at present [30]. A best example of radiotracer used in central nervous system is 2-deoxy-2-[^18^F]fluoro-D-glucose, or ^18^F-FDG (half-life: 109 min), which is transported into cells via the GLUT transporter family. It is taken up by metabolically active cells, and once intracellular, ^18^F-FDG will be phosphorylated to ^18^F-FDG-6-phosphate by hexokinase. ^18^F-based tracer has been widely used for tracking neural stem cells (Figure 2(c)) [6]. A novel agent, 3′-deoxy-3′-[^18^F]fluoro-L-thymidine, has been used for noninvasive imaging of tumor cell and NSC proliferation with PET in the previous studies [31].

However, also some obstacles were involved in the direct imaging, for example, the leakage of radiotracers into tissue cells, dilution of signal due to cell proliferation, and lack of ability to detect cell viability and function. Specially, it is crucial to identify the safe dose of a radiotracer when applying nuclear imaging with a radioisotope to the clinic treatment, taking into account the toxicity of a radiotracer. To overcome these problems is through use of indirect labelling methods. Indirect imaging of stem cells generally involves the so-called “imaging reporter genes” which is introduced into the cell's genome ex vivo. These reporter genes are able to produce the particular protein which will act with radioactive probe so that the probe signal can be detected by PET/SPECT for a long time without being limited to the half-life of the tracer used. The main advantage of reporter gene approaches is that only living cells will be identified, because only viable cells can translate the gene into a particular protein that can be acted with radioactive probe. Unlike direct labelling of cells, the reporter gene in a parent cell will be inherited to daughter cells; therefore, the tracer will not be diluted as cells divide. Additionally, when the transplanted cells die, the imaging signal will be lost, avoiding the false signal [32]. However, the use of reporter genes in human cell therapy still remains limited because whether the introduction of reporter genes into the host cell genome will cause detrimental effects or not is unknown.

### 1.3. Optical Imaging

Compared with MRI and nuclear imaging for tracking stem cells, optical imaging has advantages of lower cost, rapid acquisition, no radiation toxicity, and relatively high sensitivity (Figure 1()) [33]. Fluorescence imaging has been served in the field of cell therapy for CNS disorders for many years, using green fluorescent protein (GFP) and red fluorescent protein (RFP), as well as some fluorescent dyes such as DiD, Dil, and indocyanine Green (ICG) [5]. However, the application of fluorescence-based imaging techniques in cell tracking is limited by the short wavelengths as it is unable to obtain fluorescence signal through the bone and skin [34]. On the other hand, semiconductor nanocrystals, also called quantum dots (QDs), are a novel class of biocompatible fluorescent that are relatively photostable and have narrow luminescence bands used in cell tracking. Near-infrared- (NIR-) emitting QDs may be especially useful to track transplanted cells in the human brain because their longer wavelengths allow easier penetration of tissue such as bone and skin [35]. Many studies demonstrate the safety and efficacy of NIR fluorescence labeling with QDs as a method of identifying and tracking stem cells in a rodent model of cerebral infarction. NIR fluorescence labeling allows noninvasively tracking of transplanted cells engrafted in the infarction region as long as 8 weeks after transplantation [36]. Recently, a study of injecting embryonic stem cells labeled with six different QDs into mice backs showed QD800-labeled cells providing most prominent fluorescence intensity [37]. Those findings suggest that NIR fluorescence imaging is a long-term, noninvasive imaging technology in the field of cell therapy in vivo. Therefore, NIR-emitting tracer may be a potential tool to track the transplanted cells in humans.

However, cell labeling with QDs also could not image transplanted cells for a long time as directly labeling with regard to dilution due to cell proliferation. Additionally, when used for biological imaging and cell therapy, the toxicity of QD limits its wide usefulness. However, thanks to the recent advances in the development of surface coating material, more biocompatible QDs were used in cell tracking. In a recent study by Chen et al., cells labeled with Ag_2_S QDs were transplanted into a mice model to visualize cell dynamic migration. The difference of cell viability, proliferation, and the pluripotency-associated transcription factors released by stem cells is negligible between control and labeled hMSCs [38].

Bioluminescence imaging (BLI) has been widely applied in preclinical studies of stem cell imaging to in the brain for years. Bioluminescence involves introducing a reporter gene, which could code for a special luciferase protein, into the target stem cells. The charge-coupled device camera system can detect and quantify the photons emitted through the progress of the luciferase enzyme reacting with its substrate luciferin or coelenterazine [39]. Luciferase transformed d-luciferin into oxy-luciferin and light at the present with ATP and O_2_ in order for signals to be detected. In addition, BIL could be used to quantify the number of transplanted cells as the light emission is directly proportional to the number of cells [40]. Bioluminescence can track stem cells for a considerable long term due to the luciferase gene that is stably integrated into the genome of stem cells. Therefore, BLI also was used to study gene expression quantification, tumor development tracking in rats, and stem cell localization in mice (Figure 2(d)) [41]. However, at present, BIL is only confined to small animals, but not to large animals, because BIL can only penetrate a few centimeters of tissue. Moreover, the introduction of a reporter gene runs imponderable risk for the clinic application. Therefore, BLI is limited for a preclinical study.

### 1.4. Multimodality

As described above, no single imaging technologies can provide all the information required in tracking stem cells and monitoring their biological behavior; therefore, researchers tried to develop multimodality image to overcome the drawbacks of single imaging technology. Multimodality molecular imaging generally combined more than one imaging modality with the purpose of integrating modality-specific strengths [42, 43]. For example, a complementary use of SPECT for high indication of functional activity and CT for anatomic images enables the integration of structural and functional information, which has been used in clinic for many years.

Multimodality noninvasive imaging reporter genes can now also be developed to be combined with different imaging technologies to obtain sufficient information of the biologic behavior of stem cells. The widely employed strategies of multimodal reporter gene imaging are as follows: incorporate more than one reporter gene into one plasmid; incubate the plasmid and stem cells in order to facilitate plasmid to “enter” the cells; those genes are then transcribed into different proteins which can be imaged by different imaging modalities. In one study of Jackson et al., they used USPIO-MRI and ^11^C PET to monitor stem cell viability, proliferation, and differentiation in an animal model of Parkinson's disease for the first time. They combined the advantage of high anatomical spatial resolution in MRI and high sensitivity in PET to obtain sufficient information to assess dopaminergic function [43]. Additionally, BIL/PET imaging was deemed feasible by Cao et al. [44] and Waerzeggers et al. [45] using reporter gene technology which BLI served the higher sensitivity for detecting luc-expressing cells and ^18^F–FHBG-PET served for localization of tk-expressing cells.

Although multimodality noninvasive imaging has been successfully used in many preclinical trials, it also has some limitations. As fusion proteins containing different types of molecular probes or substrates are needed for multimodality imaging, fusion reporter genes generally are difficult to construct with a large size. Additionally, fusion proteins may lose some bioactivity at the process of gene fusion and protein expression. Therefore, it is necessary to develop a signal molecular probe or reporter gene available for multimodal imaging. A single reporter gene, Human TYR, can be detected and imaged by photoacoustic imaging, MRI, and PET in vivo and may overcome some of the aforementioned limitations. This system combines the high sensitivity for both PAI and PET and high spatial resolution for T1-weighted images, which may be a potential tool in biomedical research [32].

Another type of multimodality imaging is based on multimodal contrast agents, which integrate multiple properties in one agent to be detected by several imaging techniques. Magnetic quantum dots which combine fluorescent QDs with magnetic nanoparticles form a novel type of new materials for bioimaging. As the fluorescence and magnetic properties are integrated in a single agent, the advantage of fluorescence image and MRI can be combined to obtain the required information of transplanted cells [46]. As described before, Mn is the common useful T1 contrast agent used for cell tracking. Radiomanganese (^51^Mn and ^52^Mn) was ever used as a myocardial perfusion PET agent, with successful studies conducted in humans. Of which, ^52^Mn (t1/2 = 5.591D) has presented itself as a strong candidate for PET applications. Therefore, ^52^Mn-based PET not only could offer high sensitivity and reduced manganese dose, but also provides valuable complementary information paired with manganese-enhanced MRI (MEMRI). Importantly, besides cell tracking in the central neural system, this dual-modality manganese-based PET/MRI approach may be used to other aspects, including neuronal tract tracing and brain activation-induced uptake measurement [47].

### 1.5. Limitation

In spite of these successes and great potentials, many problems exist in these cell tracking technologies, including cytotoxicity, signal dilution, or loss in long-term tracking due to cell proliferation, insufficiency of single imaging technology to attain comprehensive information of cell dynamic state, and limited capability of revealing cell functionality and viability [48]. It is necessary to overcome these problems before cell therapy applied for clinic treatments. Currently, there is no perfect tracking agent approved by FDA to label and track stem cells for the purpose of cell therapy. It is important to understand whether those tracking agent affect the viability, differentiation, migration/homing, distribution, and engraftment of stem cells before their applications in the clinics. Many factors including composition, particulate shape, appropriate size, and surface functional groups are related to the cytotoxicity of tracking agent. Specially, different studies have different views on the cytotoxicity of the signal tracking agent. For example, SPIOs are generally considered as nontoxic in most studies; however, SPIOs coated with poly-L-lysine were reported to partially impair the differentiation function and potentials of some stem cells. As pointed out, for now, manganese and gadolinium are unlikely to be used clinically because of their metal toxicity. One of the most critical issues in stem cell therapy is how to trace and monitor the transplanted cells in vitro for a long enough time. The rapid increase in the number of transplanted stem cells limits the use of MRI agents or radiotracer, which leads to the dilution or deletion of labeled tracers. Additionally, although multiple modalities over single modality may attain more necessary information to reveal the spatial location of transplanted cells, many problems, such as more equipment and cost and higher technical difficulty, must be overcome. As a point before, fusion reporter genes, which can be detected by MRI and PET simultaneously, are usually larger and difficult to construct. Thus, the best solution is to construct a single reporter gene that can be detected by multiple imaging methods.

In the previous studies, the small rodent models are highly useful for stem cell preclinical experiment. However, the small rodent central nervous system and cerebrovasculature are different from those of human, which limits the transformation of the result of animal research into prospective clinic application directly. Large animal models may short the gap between rodent animals and humans to a certain extent, which have been used in lab but were complicated and expensive. Furthermore, clinical imaging has more limitation compared with experimental animal studies, for example, animal MRI scanners can reach16T or higher, whereas high field in human studies is around 7T, as most clinical MRI scanners being less than 3T in the country. More importantly, currently tracking technologies can only provide the certain information of migration routine and final temporal-spatial location of transplanted stem cells. For clinical researchers, it is more meaningful to visualize the viability and differentiation of transplanted stem cells and even cell functionality. One approach is to design an advance nanoparticle probe, which can detect stimuli associated with stem cell viability or functionality. Those stimuli include growth factors and enzymes expressed by stem cells, chemical secretion during cell differentiation, transgene expression during cell growth, intercellular and extracellular pH changes during cell death, and metal ion level which is essential for cell to play its normal physiological function.

## 2. Conclusion and Future Prospects

Stem cell therapies based on animal model have provided much evidence of benefits for neurological diseases. However, unless safety and efficacy of the transplanted cells are guaranteed, stem cell therapy can be taken to the clinical trial. Therefore, it is important to track the biological behaviors of transplanted cells in vivo, including proliferation, migration, viability, and functional reconstruction. Currently, every imaging technique used for cell tracking has merits and defects. The selection of imaging methods and tools should accord to the requirements and designs of the study: is high sensitivity or high spatial resolution or low cost needed? Among the various molecular imaging approaches mentioned above, MRI is the most promising tool for use in the clinic, since it is nonradioactive and not hampered by tissue depth. However, more data could be gotten to present a clearer sight of survival, differentiation, and migration routine of the transplanted stem cells in the host, through combining different imaging techniques such as PET, SPECT, and optical imaging. Furthermore, multimodality imaging strategy may overcome the instinctive drawbacks of signal imaging modality, as the combination of two or more imaging modalities may provide more comprehensive information for clinical setting. More importantly, advance imaging modalities which can reveal the viability, differentiation, distribution, and function reconstruction of transplanted cells would greatly promote the clinical application of stem cell therapy in the future.

## Figures and Tables

**Table 1:** Imaging modalities currently available for tracking neural stem cells.

Modality	Source of imaging	Type of probe	Spatial resolution	Temporal resolution	Tissue penetrating depth	Sensitivity	Clinical use	Advantages	Disadvantages
*MRI*									
In vivo labeling	Radiowave	Para- (Gd3+/Mn2+), SPIO or ^19^F	>25 *μ*m	Min–hrs	No limit	mM–*μ*M	Yes	No radiation, very good tissue contrast, high resolution	Low sensitivity, agent dilution
Ex vivo labeling	Radiowave	MR reporter genes						Long-term imaging, long-term imaging	Exogenous gene risk
*PET*									
Direct labeling	High-energy *γ*-ray	Radionuclides (e.g., ^18^F, ^11^C)	>1 mm	Sec–min	No limit	pM	Yes	High sensitivity, high sensitivity, deep tissues	Radiation, radiotracer dilution
Indirect labeling	High-energy *γ*-ray	Reporter genes (e.g., HSV1-tk)						Long-term imaging, avoid false signal, nontoxicity	Exogenous gene risk
*SPECT*									
Direct labeling	Low-energy *γ*-ray	Radionuclides (e.g., ^111^In, ^99^mTc)	>1 mm	Min	No limit	pM	Yes	High sensitivity, able to image deep tissues	Radiation, low resolution, radiotracer dilution
Indirect labeling	Low-energy *γ*-ray	Reporter genes						Long-term imaging, nontoxicity	Exogenous gene risk
*Optical imaging*									
Fluorescence imaging	Visible light	Fluorescence near-infrared dye, QD light	>2 mm	Sec–min	<1 cm	nM-pM	No	Cheap, simple, high sensitivity, activatable	Deep tissue limited, low resolution, tissue damaging
BIL	Visible light	Reporter genes	>2 mm	Sec–min	<1 cm	nM	No	Simple, high sensitivity	Deep tissue limited, low resolution
